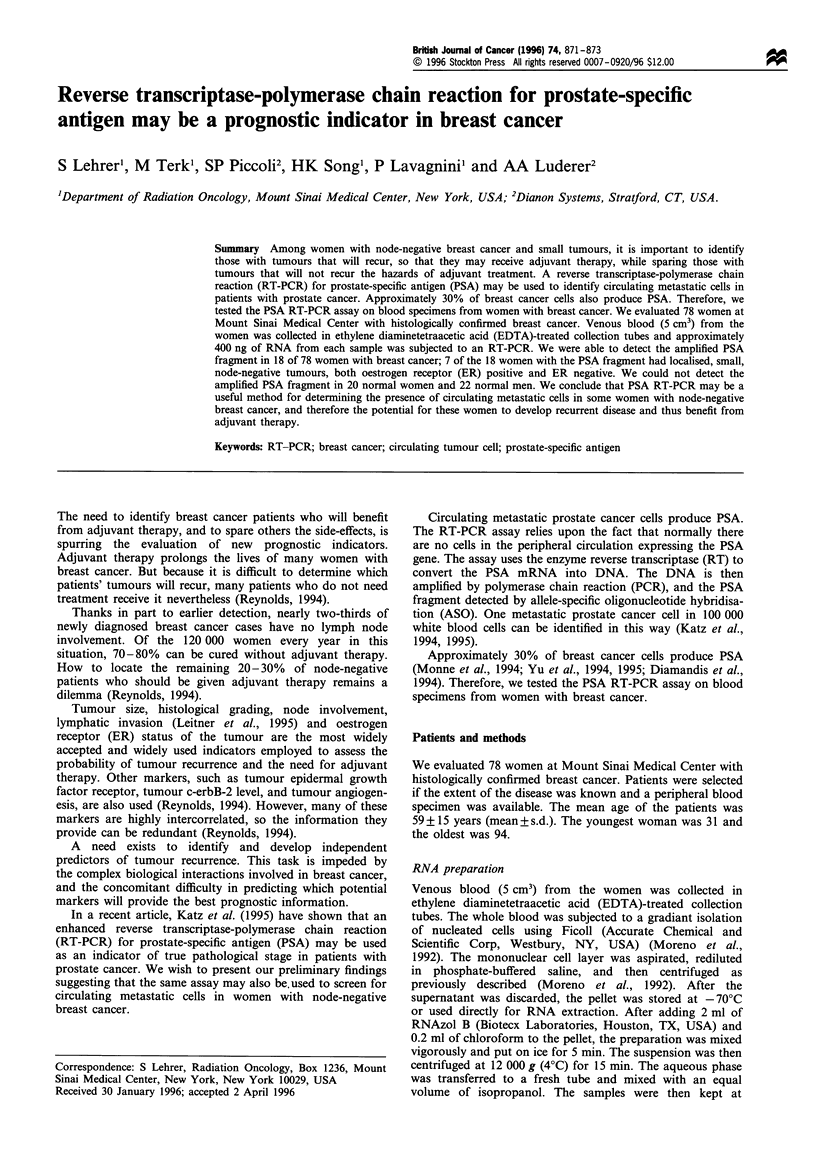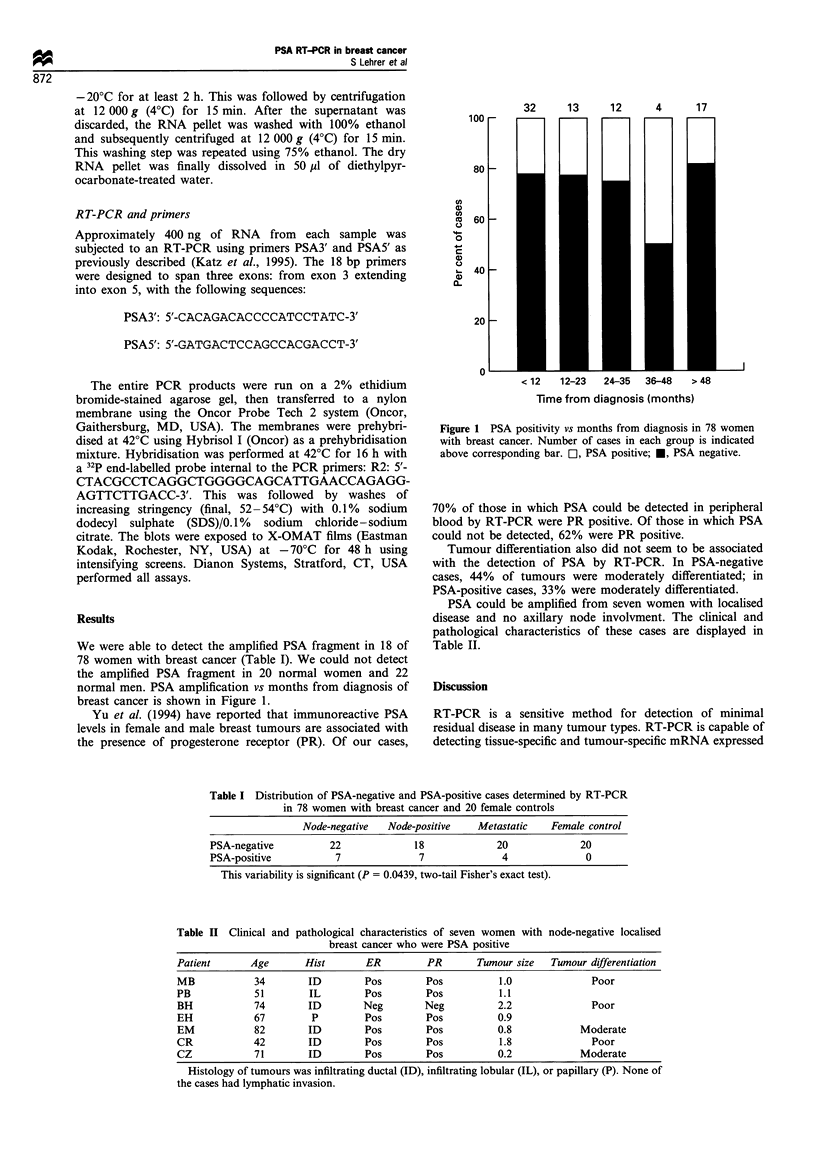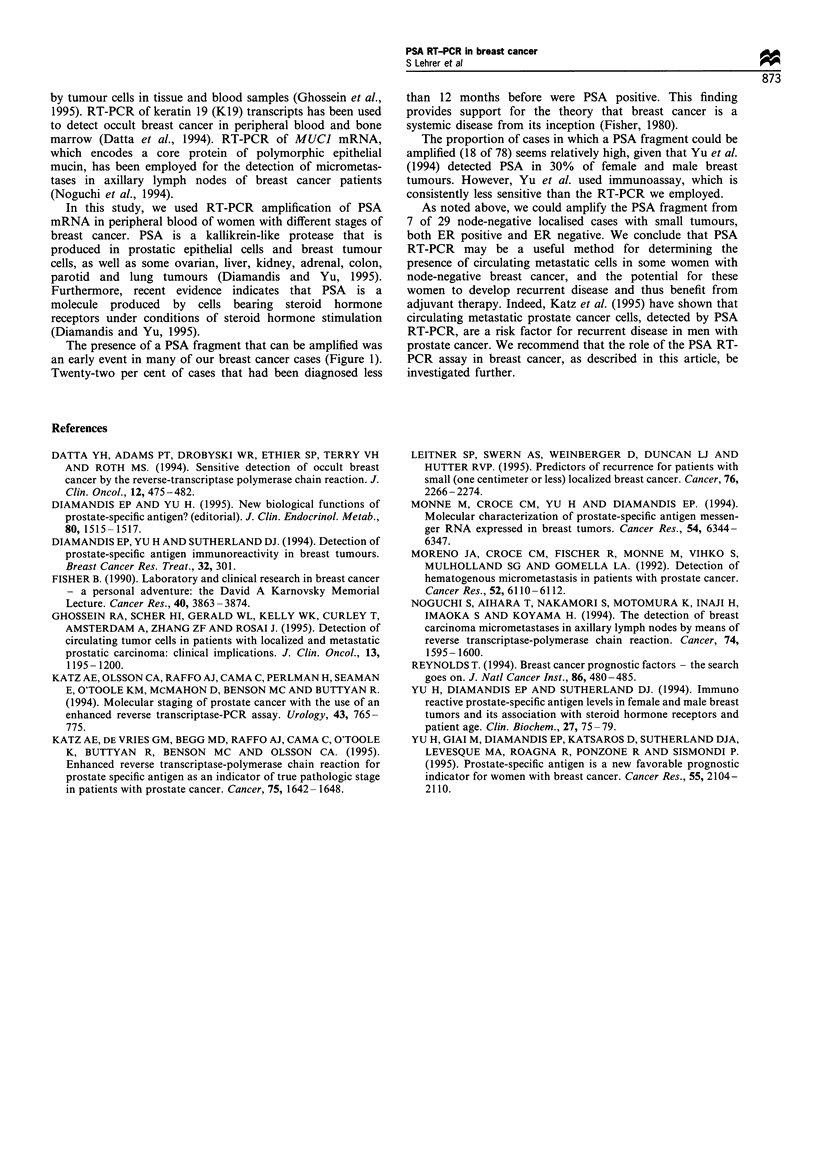# Reverse transcriptase-polymerase chain reaction for prostate-specific antigen may be a prognostic indicator in breast cancer.

**DOI:** 10.1038/bjc.1996.450

**Published:** 1996-09

**Authors:** S. Lehrer, M. Terk, S. P. Piccoli, H. K. Song, P. Lavagnini, A. A. Luderer

**Affiliations:** Department of Radiation Oncology, Mount Sinai Medical Center, New York, USA.

## Abstract

Among women with node-negative breast cancer and small tumours, it is important to identify those with tumours that will recur, so that they may receive adjuvant therapy, while sparing those with tumours that will not recur the hazards of adjuvant treatment. A reverse transcriptase-polymerase chain reaction (RT-PCR) for prostate-specific antigen (PSA) may be used to identify circulating metastatic cells in patients with prostate cancer. Approximately 30% of breast cancer cells also produce PSA. Therefore, we tested the PSA RT-PCR assay on blood specimens from women with breast cancer. We evaluated 78 women at Mount Sinai Medical Center with histologically confirmed breast cancer. Venous blood (5 cm3) from the women was collected in ethylene diaminetetraacetic acid (EDTA)-treated collection tubes and approximately 400 ng of RNA from each sample was subjected to an RT-PCR. We were able to detect the amplified PSA fragment in 18 of 78 women with breast cancer; 7 of the 18 women with the PSA fragment had localised, small, node-negative tumours, both oestrogen receptor (ER) positive and ER negative. We could not detect the amplified PSA fragment in 20 normal women and 22 normal men. We conclude that PSA RT-PCR may be a useful method for determining the presence of circulating metastatic cells in some women with node-negative breast cancer, and therefore the potential for these women to develop recurrent disease and thus benefit from adjuvant therapy.


British Journal of Cancer (1996) 74, 871-873
© 1996 Stockton Press All rights reserved 0007-0920/96 $12.00

### Reverse transcriptase-polymerase chain reaction for prostate-specific antigen may be a prognostic indicator in breast cancer

S Lehrer[1], M Terk[1], SP Piccoli[2], HK Song[1], P Lavagnini[1] and AA Luderer[2]

[1]*Department of Radiation Oncology, Mount Sinai Medical Center, New York, USA;* [2]*Dianon Systems, Stratford, CT, USA.*

**Summary** Among women with node-negative breast cancer and small tumours, it is important to identify those with tumours that will recur, so that they may receive adjuvant therapy, while sparing those with tumours that will not recur the hazards of adjuvant treatment. A reverse transcriptase-polymerase chain reaction (RT-PCR) for prostate-specific antigen (PSA) may be used to identify circulating metastatic cells in patients with prostate cancer. Approximately 30% of breast cancer cells also produce PSA. Therefore, we tested the PSA RT-PCR assay on blood specimens from women with breast cancer. We evaluated 78 women at Mount Sinai Medical Center with histologically confirmed breast cancer. Venous blood (5 $cm^3$) from the women was collected in ethylene diaminetetraacetic acid (EDTA)-treated collection tubes and approximately 400 ng of RNA from each sample was subjected to an RT-PCR. We were able to detect the amplified PSA fragment in 18 of 78 women with breast cancer; 7 of the 18 women with the PSA fragment had localised, small, node-negative tumours, both oestrogen receptor (ER) positive and ER negative. We could not detect the amplified PSA fragment in 20 normal women and 22 normal men. We conclude that PSA RT-PCR may be a useful method for determining the presence of circulating metastatic cells in some women with node-negative breast cancer, and therefore the potential for these women to develop recurrent disease and thus benefit from adjuvant therapy.

**Keywords:** RT–PCR; breast cancer; circulating tumour cell; prostate-specific antigen

The need to identify breast cancer patients who will benefit from adjuvant therapy, and to spare others the side-effects, is spurring the evaluation of new prognostic indicators. Adjuvant therapy prolongs the lives of many women with breast cancer. But because it is difficult to determine which patients' tumours will recur, many patients who do not need treatment receive it nevertheless (Reynolds, 1994).

Thanks in part to earlier detection, nearly two-thirds of newly diagnosed breast cancer cases have no lymph node involvement. Of the 120 000 women every year in this situation, 70–80% can be cured without adjuvant therapy. How to locate the remaining 20–30% of node-negative patients who should be given adjuvant therapy remains a dilemma (Reynolds, 1994).

Tumour size, histological grading, node involvement, lymphatic invasion (Leitner *et al.*, 1995) and oestrogen receptor (ER) status of the tumour are the most widely accepted and widely used indicators employed to assess the probability of tumour recurrence and the need for adjuvant therapy. Other markers, such as tumour epidermal growth factor receptor, tumour c-erbB-2 level, and tumour angiogenesis, are also used (Reynolds, 1994). However, many of these markers are highly intercorrelated, so the information they provide can be redundant (Reynolds, 1994).

A need exists to identify and develop independent predictors of tumour recurrence. This task is impeded by the complex biological interactions involved in breast cancer, and the concomitant difficulty in predicting which potential markers will provide the best prognostic information.

In a recent article, Katz *et al.* (1995) have shown that an enhanced reverse transcriptase-polymerase chain reaction (RT-PCR) for prostate-specific antigen (PSA) may be used as an indicator of true pathological stage in patients with prostate cancer. We wish to present our preliminary findings suggesting that the same assay may also be used to screen for circulating metastatic cells in women with node-negative breast cancer.

Correspondence: S Lehrer, Radiation Oncology, Box 1236, Mount Sinai Medical Center, New York, New York 10029, USA
Received 30 January 1996; accepted 2 April 1996

Circulating metastatic prostate cancer cells produce PSA. The RT-PCR assay relies upon the fact that normally there are no cells in the peripheral circulation expressing the PSA gene. The assay uses the enzyme reverse transcriptase (RT) to convert the PSA mRNA into DNA. The DNA is then amplified by polymerase chain reaction (PCR), and the PSA fragment detected by allele-specific oligonucleotide hybridisation (ASO). One metastatic prostate cancer cell in 100 000 white blood cells can be identified in this way (Katz *et al.*, 1994, 1995).

Approximately 30% of breast cancer cells produce PSA (Monne *et al.*, 1994; Yu *et al.*, 1994, 1995; Diamandis *et al.*, 1994). Therefore, we tested the PSA RT-PCR assay on blood specimens from women with breast cancer.

### Patients and methods

We evaluated 78 women at Mount Sinai Medical Center with histologically confirmed breast cancer. Patients were selected if the extent of the disease was known and a peripheral blood specimen was available. The mean age of the patients was 59 ± 15 years (mean ± s.d.). The youngest woman was 31 and the oldest was 94.

### *RNA preparation*

Venous blood (5 $cm^3$) from the women was collected in ethylene diaminetetraacetic acid (EDTA)-treated collection tubes. The whole blood was subjected to a gradiant isolation of nucleated cells using Ficoll (Accurate Chemical and Scientific Corp, Westbury, NY, USA) (Moreno *et al.*, 1992). The mononuclear cell layer was aspirated, rediluted in phosphate-buffered saline, and then centrifuged as previously described (Moreno *et al.*, 1992). After the supernatant was discarded, the pellet was stored at −70°C or used directly for RNA extraction. After adding 2 ml of RNAzol B (Biotecx Laboratories, Houston, TX, USA) and 0.2 ml of chloroform to the pellet, the preparation was mixed vigorously and put on ice for 5 min. The suspension was then centrifuged at 12 000 *g* (4°C) for 15 min. The aqueous phase was transferred to a fresh tube and mixed with an equal volume of isopropanol. The samples were then kept at

−20°C for at least 2 h. This was followed by centrifugation at 12 000 *g* (4°C) for 15 min. After the supernatant was discarded, the RNA pellet was washed with 100% ethanol and subsequently centrifuged at 12 000 *g* (4°C) for 15 min. This washing step was repeated using 75% ethanol. The dry RNA pellet was finally dissolved in 50 μl of diethylpyrocarbonate-treated water.

*RT-PCR and primers*

Approximately 400 ng of RNA from each sample was subjected to an RT-PCR using primers PSA3′ and PSA5′ as previously described (Katz *et al.*, 1995). The 18 bp primers were designed to span three exons: from exon 3 extending into exon 5, with the following sequences:

PSA3′: 5′-CACAGACACCCCATCCTATC-3′

PSA5′: 5′-GATGACTCCAGCCACGACCT-3′

The entire PCR products were run on a 2% ethidium bromide-stained agarose gel, then transferred to a nylon membrane using the Oncor Probe Tech 2 system (Oncor, Gaithersburg, MD, USA). The membranes were prehybridised at 42°C using Hybrisol I (Oncor) as a prehybridisation mixture. Hybridisation was performed at 42°C for 16 h with a $^{32}P$ end-labelled probe internal to the PCR primers: R2: 5′-CTACGCCTCAGGCTGGGGCAGCATTGAACCAGAGG-AGTTCTTGACC-3′. This was followed by washes of increasing stringency (final, 52−54°C) with 0.1% sodium dodecyl sulphate (SDS)/0.1% sodium chloride−sodium citrate. The blots were exposed to X-OMAT films (Eastman Kodak, Rochester, NY, USA) at −70°C for 48 h using intensifying screens. Dianon Systems, Stratford, CT, USA performed all assays.

### Results

We were able to detect the amplified PSA fragment in 18 of 78 women with breast cancer (Table I). We could not detect the amplified PSA fragment in 20 normal women and 22 normal men. PSA amplification *vs* months from diagnosis of breast cancer is shown in Figure 1.

Yu *et al.* (1994) have reported that immunoreactive PSA levels in female and male breast tumours are associated with the presence of progesterone receptor (PR). Of our cases,

**Figure 1** PSA positivity *vs* months from diagnosis in 78 women with breast cancer. Number of cases in each group is indicated above corresponding bar. □, PSA positive; ■, PSA negative.

70% of those in which PSA could be detected in peripheral blood by RT-PCR were PR positive. Of those in which PSA could not be detected, 62% were PR positive.

Tumour differentiation also did not seem to be associated with the detection of PSA by RT-PCR. In PSA-negative cases, 44% of tumours were moderately differentiated; in PSA-positive cases, 33% were moderately differentiated.

PSA could be amplified from seven women with localised disease and no axillary node involvment. The clinical and pathological characteristics of these cases are displayed in Table II.

### Discussion

RT-PCR is a sensitive method for detection of minimal residual disease in many tumour types. RT-PCR is capable of detecting tissue-specific and tumour-specific mRNA expressed

**Table I** Distribution of PSA-negative and PSA-positive cases determined by RT-PCR in 78 women with breast cancer and 20 female controls

| | *Node-negative* | *Node-positive* | *Metastatic* | *Female control* |
|---|---|---|---|---|
| PSA-negative | 22 | 18 | 20 | 20 |
| PSA-positive | 7 | 7 | 4 | 0 |

This variability is significant ($P$ = 0.0439, two-tail Fisher's exact test).

**Table II** Clinical and pathological characteristics of seven women with node-negative localised breast cancer who were PSA positive

| *Patient* | *Age* | *Hist* | *ER* | *PR* | *Tumour size* | *Tumour differentiation* |
|---|---|---|---|---|---|---|
| MB | 34 | ID | Pos | Pos | 1.0 | Poor |
| PB | 51 | IL | Pos | Pos | 1.1 | |
| BH | 74 | ID | Neg | Neg | 2.2 | Poor |
| EH | 67 | P | Pos | Pos | 0.9 | |
| EM | 82 | ID | Pos | Pos | 0.8 | Moderate |
| CR | 42 | ID | Pos | Pos | 1.8 | Poor |
| CZ | 71 | ID | Pos | Pos | 0.2 | Moderate |

Histology of tumours was infiltrating ductal (ID), infiltrating lobular (IL), or papillary (P). None of the cases had lymphatic invasion.

by tumour cells in tissue and blood samples (Ghossein *et al.*, 1995). RT-PCR of keratin 19 (K19) transcripts has been used to detect occult breast cancer in peripheral blood and bone marrow (Datta *et al.*, 1994). RT-PCR of *MUC1* mRNA, which encodes a core protein of polymorphic epithelial mucin, has been employed for the detection of micrometastases in axillary lymph nodes of breast cancer patients (Noguchi *et al.*, 1994).

In this study, we used RT-PCR amplification of PSA mRNA in peripheral blood of women with different stages of breast cancer. PSA is a kallikrein-like protease that is produced in prostatic epithelial cells and breast tumour cells, as well as some ovarian, liver, kidney, adrenal, colon, parotid and lung tumours (Diamandis and Yu, 1995). Furthermore, recent evidence indicates that PSA is a molecule produced by cells bearing steroid hormone receptors under conditions of steroid hormone stimulation (Diamandis and Yu, 1995).

The presence of a PSA fragment that can be amplified was an early event in many of our breast cancer cases (Figure 1). Twenty-two per cent of cases that had been diagnosed less than 12 months before were PSA positive. This finding provides support for the theory that breast cancer is a systemic disease from its inception (Fisher, 1980).

The proportion of cases in which a PSA fragment could be amplified (18 of 78) seems relatively high, given that Yu *et al.* (1994) detected PSA in 30% of female and male breast tumours. However, Yu *et al.* used immunoassay, which is consistently less sensitive than the RT-PCR we employed.

As noted above, we could amplify the PSA fragment from 7 of 29 node-negative localised cases with small tumours, both ER positive and ER negative. We conclude that PSA RT-PCR may be a useful method for determining the presence of circulating metastatic cells in some women with node-negative breast cancer, and the potential for these women to develop recurrent disease and thus benefit from adjuvant therapy. Indeed, Katz *et al.* (1995) have shown that circulating metastatic prostate cancer cells, detected by PSA RT-PCR, are a risk factor for recurrent disease in men with prostate cancer. We recommend that the role of the PSA RT-PCR assay in breast cancer, as described in this article, be investigated further.

### References

DATTA YH, ADAMS PT, DROBYSKI WR, ETHIER SP, TERRY VH AND ROTH MS. (1994). Sensitive detection of occult breast cancer by the reverse-transcriptase polymerase chain reaction. *J. Clin. Oncol.*, **12**, 475–482.

DIAMANDIS EP AND YU H. (1995). New biological functions of prostate-specific antigen? (editorial). *J. Clin. Endocrinol. Metab.*, **80**, 1515–1517.

DIAMANDIS EP, YU H AND SUTHERLAND DJ. (1994). Detection of prostate-specific antigen immunoreactivity in breast tumours. *Breast Cancer Res. Treat.*, **32**, 301.

FISHER B. (1990). Laboratory and clinical research in breast cancer – a personal adventure: the David A Karnovsky Memorial Lecture. *Cancer Res.*, **40**, 3863–3874.

GHOSSEIN RA, SCHER HI, GERALD WL, KELLY WK, CURLEY T, AMSTERDAM A, ZHANG ZF AND ROSAI J. (1995). Detection of circulating tumor cells in patients with localized and metastatic prostatic carcinoma: clinical implications. *J. Clin. Oncol.*, **13**, 1195–1200.

KATZ AE, OLSSON CA, RAFFO AJ, CAMA C, PERLMAN H, SEAMAN E, O'TOOLE KM, MCMAHON D, BENSON MC AND BUTTYAN R. (1994). Molecular staging of prostate cancer with the use of an enhanced reverse transcriptase-PCR assay. *Urology*, **43**, 765–775.

KATZ AE, DE VRIES GM, BEGG MD, RAFFO AJ, CAMA C, O'TOOLE K, BUTTYAN R, BENSON MC AND OLSSON CA. (1995). Enhanced reverse transcriptase-polymerase chain reaction for prostate specific antigen as an indicator of true pathologic stage in patients with prostate cancer. *Cancer*, **75**, 1642–1648.

LEITNER SP, SWERN AS, WEINBERGER D, DUNCAN LJ AND HUTTER RVP. (1995). Predictors of recurrence for patients with small (one centimeter or less) localized breast cancer. *Cancer*, **76**, 2266–2274.

MONNE M, CROCE CM, YU H AND DIAMANDIS EP. (1994). Molecular characterization of prostate-specific antigen messenger RNA expressed in breast tumors. *Cancer Res.*, **54**, 6344–6347.

MORENO JA, CROCE CM, FISCHER R, MONNE M, VIHKO S, MULHOLLAND SG AND GOMELLA LA. (1992). Detection of hematogenous micrometastasis in patients with prostate cancer. *Cancer Res.*, **52**, 6110–6112.

NOGUCHI S, AIHARA T, NAKAMORI S, MOTOMURA K, INAJI H, IMAOKA S AND KOYAMA H. (1994). The detection of breast carcinoma micrometastases in axillary lymph nodes by means of reverse transcriptase-polymerase chain reaction. *Cancer*, **74**, 1595–1600.

REYNOLDS T. (1994). Breast cancer prognostic factors – the search goes on. *J. Natl Cancer Inst.*, **86**, 480–485.

YU H, DIAMANDIS EP AND SUTHERLAND DJ. (1994). Immuno reactive prostate-specific antigen levels in female and male breast tumors and its association with steroid hormone receptors and patient age. *Clin. Biochem.*, **27**, 75–79.

YU H, GIAI M, DIAMANDIS EP, KATSAROS D, SUTHERLAND DJA, LEVESQUE MA, ROAGNA R, PONZONE R AND SISMONDI P. (1995). Prostate-specific antigen is a new favorable prognostic indicator for women with breast cancer. *Cancer Res.*, **55**, 2104–2110.